# Macrophages from gut-corrected CF mice express human *CFTR* and lack a pro-inflammatory phenotype

**DOI:** 10.1016/j.jcf.2021.11.004

**Published:** 2022-03

**Authors:** Jonathan L Gillan, Gareth R Hardisty, Donald J Davidson, Robert D Gray

**Affiliations:** Centre for Inflammation Research, Queen's Medical Research Institute, University of Edinburgh, 47 Little France Crescent, Edinburgh, EH16 4TJ, UK

## Abstract

Macrophages represent prominent immune orchestrators of cystic fibrosis (CF) inflammation and, as such, are an ever-increasing focus of CF research with several reports of intrinsic immune dysfunction related to loss of CFTR activity in macrophages themselves. Animal models of CF have contributed, in no small part, to a deepening of our understanding of the pathophysiology of the disease and towards therapeutic development. A commonly-used animal model in CF research is the *Cftr*^tm1Unc^ Tg(FABP-hCFTR) mouse, which displays gut-specific expression of a human *CFTR* transgene in order to rescue the high rate of early mortality in *Cftr*-null mice associated with severe intestinal obstruction. We find significant variation in the response to inflammatory challenge of patient macrophages and cells derived from the *Cftr*^tm1Unc^ Tg(FABP-hCFTR) mouse and show that macrophages derived from this mouse exhibit aberrant expression of human *CFTR*. This may contribute to the absence of inflammatory changes in this model.

## Highlights

• The *cftr*^tm1unc^ Tg(FABP-hCFTR) mouse is a commonly-used animal model of CF.
• This mouse expresses human *CFTR* in the gut to prevent fatal intestinal obstruction.
• Macrophages from this mouse fail to replicate immune dysfunction seen in patient cells.
• We show ectopic expression of human *CFTR* transgene in macrophages from this CF mouse.
• This may help to explain anomalies in the field related to use of this model.

## 1 Introduction

Chronic airway inflammation underpins the progression of CF lung disease and is consequently a major focus of CF research and an important therapeutic target. There is ample evidence of dysregulated inflammatory pathways in immune cells derived from people with CF and the observation of functional CFTR expression in non-epithelial cells has uncovered a direct role for the basic defect in immune cell dysfunction in CF, particularly in monocytes and macrophages [1], [2], [3]. Rollout of disease-modifying CFTR modulators to the vast majority of people with CF may now place further emphasis on animal models to provide a means of investigating baseline differences in uncorrected, CFTR-deficient immune cells with the aim of developing novel strategies to combat chronic inflammation alongside modulator therapies and the standard treatment regimen [4]. Identification of the *CFTR* gene in the 1980s allowed for the generation of animal models and heralded a new wave of CF *in vivo* research which has helped considerably to inform a greater understanding of the pathophysiology of the disease. The mouse is the most commonly used model of CF and has many advantages, not least the ease of husbandry and low-cost relative to larger animals which are more anatomically homologous to humans (e.g. pig and ferret). However, CF mice fail to exhibit several of the most prominent hallmarks of human disease and do not develop mucus obstruction and spontaneous lung disease to anywhere near the same extent as humans with CF [5–7]. In addition, complete knockout of *Cftr* leads to high rates of early mortality in these models due to severe intestinal obstruction [8]. One CF mouse model designed specifically to offset this issue is the *Cftr*^tm1Unc^ Tg(FABP-hCFTR) mouse which displays gut-specific expression of a human CFTR transgene, driven by the intestinal fatty acid-binding protein 2 (FABP2) promoter [9]. This allows for survival to maturity with correction of the CF phenotype restricted largely to the gut and this model is subsequently in widespread use in studying the effects of CFTR loss on other systems [10–14].

Here, we show that bone-marrow derived macrophages (BMDMs) from the *Cftr*^tm1Unc^ Tg(FABP-hCFTR) mouse display significant differences to patient-derived monocyte-derived macrophages (MDMs) in response to inflammatory challenge. We then demonstrate expression of the human *CFTR* transgene in BMDMs from the same mouse model. Together these data suggest that this widely used mouse model has significant limitations in the study of CF associated inflammation.

## 2 Materials and methods

### 2.1 Mice

*Cftr*^tm1Unc^ Tg(FABP-hCFTR)1Jaw/J were purchased from Jackson Laboratory and then backcrossed for 10 generations onto C57BL/6 J mice. Male homozygotes were bred with female heterozygotes due to poor fertility in the female homozygotes, with genotyping carried out at birth to determine deletion of murine *Cftr*, and the presence of the human transgene. The mice harbour the FABP-hCFTR transgene with inserted rat fatty acid-binding protein 2 (FABP2) promoter directing expression of human CFTR allowing for high expression of the transgene throughout the small intestine [9]. Mouse breeding and experimental work was carried out in accordance with the Animal (Scientific Procedures) Act 1986, PPL number 70/8884, and under the supervision of the University of Edinburgh Ethical Review Committee. All researchers were accredited by the Home Office, UK.

### 2.2 Macrophage culture and treatment

Bone marrow-derived macrophages (BMDMs) were obtained by flushing bone marrow from femurs and tibias and plated at 3 × 10^6^ per well in 6-well plates for 7 days in Dulbecco's Modified Eagle Medium (DMEM) + glutMAX (Thermo Fisher Scientific) supplemented with 10% heat-inactivated foetal calf serum (HI-FCS), 1% penicillin/streptomycin (P/S), 1% l-glutamine (all Thermo Fisher Scientific) and 20% M-CSF-containing L929 medium to induce macrophage differentiation. After 7 days, BMDMs were detached using ice-cold cation-free Dulbecco's phosphate buffered saline (DPBS^−/−^) (Thermo Fisher Scientific) + 1% BSA (Sigma) + 3 mM EDTA (Thermo Fisher Scientific), counted and plated overnight at 0.25 × 10^6^/well on a 48-well culture plate.

Peripheral blood was collected from clinically stable patients (no intravenous antibiotics required in the 2 weeks previous to sample collection) with CF attending the Scottish National CF Service at the Edinburgh Western General Hospital. Lung transplant recipients were excluded. Written consent was given by patients and the study was approved by the East of Scotland Research Ethics Committee (15/ES/0094). All individuals recruited had at least one copy of the ΔF508 mutation with another disease-causing mutation (four ΔF508/ΔF508 homozygous, one ΔF508/1717 + 1G > A, one ΔF508/D1152H, ΔF508/G542X and one ΔF508/Q493X). Peripheral blood was also collected from age- and sex-matched healthy adult human volunteers under Ethical Review (AMREC Reference number 15-HV-013) under the project number CIRBRP009; this included written informed consent. Peripheral blood mononuclear cells (PBMCs) were isolated by Percoll (GE Healthcare) density gradient centrifugation [15]. Monocytes were isolated from the PBMCs by pan monocyte negative selection using MACS cell separation, according to manufacturer's instructions (Miltenyi Biotec). Isolated monocytes were plated at 0.8 × 10^6^/well into 24-well tissue culture plates (Sigma-Aldrich) and cultured in Iscove's Modified Dulbecco's Medium (IMDM) supplemented with 10% HI-FCS, 1% P/S, 1% l-glutamine, and 100 ng/ml human recombinant macrophage colony-stimulating factor (M-CSF; PeproTech) for 7 days to induce differentiation.

Lipopolysaccharide (LPS) challenge of human or mouse macrophages was carried out with 100 ng/ml *Pseudomonas aeruginosa* 10 LPS (Sigma-Aldrich) for the relevant time points.

### 2.3 Inflammatory gene expression and cytokine secretion analysis

RNA extraction from cell lysates was performed using the RNeasy Mini Kit (Qiagen) with on-column DNase I digestion also carried out before final elution using the RNase-Free DNase Set (Qiagen), both as per manufacturer's instructions. cDNA was synthesised using a master mix containing reverse transcriptase and other reagents from the High-Capacity cDNA Reverse Transcription Kit (Thermo Fisher Scientific) as well as RNase Inhibitor (Thermo Fisher Scientific), according to manufacturer's instructions.

Quantitative real time PCR (qRT-PCR) was carried out on StepOnePlus™ Real-Time PCR System (Applied Biosystems) using SYBR green to assess for expression of various macrophage-associated inflammatory genes. C_T_ values were obtained for each sample and expression fold change was measured using the 2^−ΔΔCt^ method with normalisation against 18S rRNA.

Supernatants of murine BMDM cultures were collected and assayed for levels of several inflammatory cytokines and chemokines using the LEGENDPlex™ Mouse Inflammation Panel cytokine bead array kit, as per manufacturer's instruction (BioLegend). Human TNF-α secretion in MDM supernatants was measured by ELISA (R&D Systems).

### 2.4 CFTR/CFTR gene and protein expression analysis

Prescence of CFTR mRNA was confirmed using gel electrophoresis and cDNA obtained from fully differentiated BMDMs, with cDNA from human bronchial epithelial cell line, 16HBE, used for human control. Sequence homology prevented design of human CFTR-specific primers and so cross-species CFTR specific primers (mouse *Cftr/*human *CFTR:* (5′−3′) Forward – GGAGAGCATACCAGCAGTGACT; Reverse - TTCCAAGGAGCCACAGCACAAC) were used in combination with mouse CFTR specific primers (mouse *Cftr*: (5′−3′) Forward – CCATCAGCAAGCTGAAAGCAGG; Reverse - GTAGGGTTGTAATGCCGAGACG). SYBR green qRT-PCR was carried out to quantify CFTR mRNA abundance between samples.

BMDMs were derived for protein analysis from femurs of mice (*Cftr*^tm1Unc^ Tg(FABP-hCFTR and WT littermate) obtained from collaborators at Imperial College London. BMDMs were culture for 7 days, as before, and lysed on ice using RIPA buffer (Thermo Fisher Scientific) supplemented with cOmplete™ Protease Inhibitor Cocktail (Roche). SDS polyacrylamide gel electrophoresis was carried out using precast 4–12% Bis-Tris gels and MOPS SDS running buffer for 90 min at 100 V. Proteins were transferred to a PVDF membrane and blocked for 1 h with TBST + 5% milk before being incubated with anti-CFTR primary antibody (1:1000; CF3; Novus Biologicals) or anti β-Actin primary antibody (1:2000; C4; Santa Cruz Biotechnology) overnight at 4 °C. HRP-linked goat anti-mouse secondary antibody (1:5000; Jackson ImmunoResearch) was then incubated with the membrane for 1 hour before exposure to chemiluminescence substrate (Thermo Fisher Scientific) was added to the membranes for 1 min before detection of chemiluminescence on x-ray film.

## 3 Results

### 3.1 Major disparities exist in the proinflammatory response of human and mouse CF macrophages to LPS challenge

**Fig 1:**
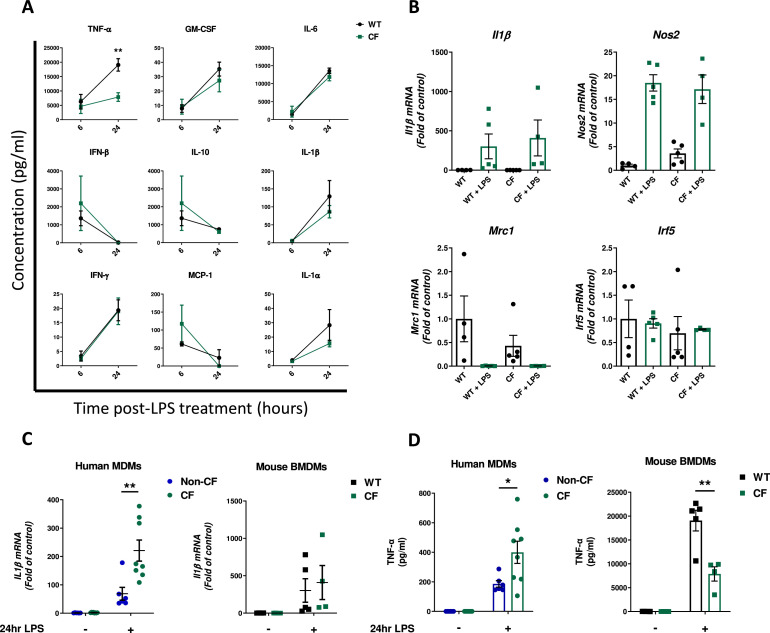
Inflammatory Phenotype in Human CF MDMs is not replicated in Macrophages Derived from *Cftr*^tm1Unc^ Tg(FABPhCFTR) Mouse. A. Secretion of various inflammatory cytokines in supernatants of WT or CF BMDMs following activation with 100 ng/ml LPS for 6 and 24 h (*n* = 5 WT and 4 CF). B. Expression of gene markers of macrophage polarisation (Classical/M1 – *Il1β, Nos2, Irf5*. Alternatively-activated/M2 – *Mrc1*) by CF BMDMs compared to WT at 24 h with or without LPS treatment (*n* = 4 WT and 4 CF). C. Comparison of human *IL1β* expression by CF and non-CF human MDMs at 24 h post LPS with murine *Il1β* expression by CF and WT BMDMs at the same time point following the same stimulation (Human *n* = 6 nonCF and 8 CF). D. Supernatant levels of human TNF-α produced by human MDMs and murine TNF-α by mouse BMDMs at 24 h post Pseudomonas LPS (Human *n* = 6 non-CF and 8 CF). * = *P* ≤ 0.05, ** = *P* ≤ 0.01. Obtained via 2-way ANOVA with Tukey's multiple comparison post-hoc test. Data are presented as mean ± SEM.

Analysis of cytokine secretion by mouse BMDMs at 6 and 24 h following treatment with *P. aeruginosa* LPS found no significant differences in release of several key inflammatory cytokines with the exception of TNF-α, secretion of which by CF macrophages was significantly reduced compared to wild-type (WT) (Fig. 1A). There were no significant differences in expression of several murine gene markers of macrophage polarisation between LPS-activated WT and CF BMDMs (Fig. 1B). The activated immune phenotype of the CF BMDMs was then compared to patient MDMs, subjected to the same LPS challenge. Expression of proinflammatory *Il1β*, as measured by qRT-PCR, remained highly upregulated after 24 stimulation with LPS compared to untreated controls but there was no significant difference in the magnitude of this upregulation between CF and WT BMDMs, however, expression of the human orthologue was significantly elevated in patient MDMs at the same time point relative to non-CF controls (Fig. 1C). Furthermore, whilst secretion of TNF-α was upregulated in LPS-treated CF MDMs at 24 h, release of the equivalent cytokine in mice was significantly blunted in activated CF BMDMs compared to WT (Fig. 1D).

### 3.2 BMDMs derived from *Cftr*^tm1unc^ tg(fabp-hcftr) mouse express human cftr transgene and cftr protein

**Fig 2:**
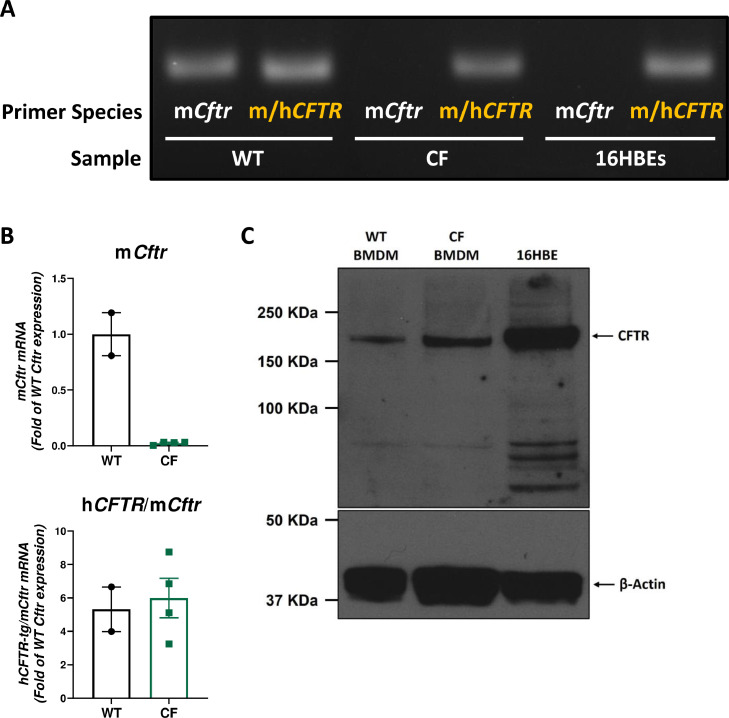
Aberrant Expression of Human CFTR Transgene in BMDMs Derived from *Cftr*^tm1Unc^ Tg(FABP-hCFTR) Mouse. A. Qualitative analysis by PCR gel electrophoresis of CFTR gene expression in WT and CF BMDMs and human bronchial epithelial cell line, 16HBEs. mCftr denotes mouse Cftr-specific transcript amplification whereas m/hCFTR primers bind both human and mouse CFTR. B. Quantitative measurement of CFTR expression in CF BMDMs by qRT-PCR. Data are presented as mean ± SEM. C. The presence of CFTR protein in lysates from BMDMs derived from Cftrtm1Unc Tg(FABP-hCFTR) mice or WT littermates and from 16HBEs was detected by western blot. β-Actin protein detection is shown as loading control.

Given that mouse BMDMs failed to display a clear functional inflammatory defect, of the nature associated with CFTR deficiency in human cells, the expression of both *Cftr* and the human *CFTR* transgene were assessed. The presence of human *CFTR* mRNA in murine CF (*Cftr*^tm1Unc^ Tg(FABP-hCFTR)) BMDMs was confirmed by gel electrophoresis following PCR using mouse/human CFTR primers in combination with mouse *Cftr*-specific primers. This showed that, as expected, WT macrophages express only murine *Cftr* and human bronchial epithelial cells (16HBEs) express only the human gene. In contrast, BMDMs derived from the *Cftr*^tm1Unc^ Tg(FABP-hCFTR) mouse express no murine *Cftr* (as expected due to the *Cftr* deletion) but do display expression of the *CFTR* transgene (Fig. 2A). This was further underlined by quantitative analysis of gene expression by qRT-PCR, showing levels of human *CFTR* expression in *Cftr*^tm1Unc^ Tg(FABP-hCFTR) BMDM that were comparable to *Cftr* expression in WT mice (Fig. 2B). Femurs were then obtained from *Cftr^tm1Unc^ Tg(FABP-hCFTR)* mice from a separate institution (Imperial College London) and utilised to generate BMDMs, as before. Western blot analysis confirmed expression of CFTR protein in BMDMs from the CF mice, as well as in WT littermates and 16HBEs (Fig. 2C).

## 4 Discussion

This study underlines key differences in the inflammatory response between macrophages from people with CF and macrophages from a commonly used murine model of CF. Inflammation is a cornerstone of CF lung disease and investigation of immune cell dysfunction in CF is now a central aspect of CF research with the aim of identifying novel ways to dampen chronic inflammation therapeutically and slow lung function decline. Macrophages represent one major target of such investigations and have been shown to display intrinsic, CFTR-dependent immune dysregulation in CF that likely contributes to exacerbation of the chronic inflammatory landscape [16]. This phenotype has been shown in various models of CF including in the *CFTR*^−/-^ pig and in mice using myeloid-specific deletion of *Cftr*, whether through use of bone marrow chimeras or LysMCre conditional models, demonstrating a role for CFTR-deficient macrophages in the aggravation of airway inflammation and thwarting of effective resolution in such mice following inflammatory or infectious challenge [17], [18], [19]. Animal models of CF that are easy to breed and analogous, in key aspects of disease manifestation, to people with CF are highly important to the field CF research. The mouse is the most commonly used model, with the *Cftr*^tm1Unc^ Tg(FABP-hCFTR) gut-correction model in widespread use. Here, we show that BMDMs derived from the *Cftr*^tm1Unc^ Tg(FABP-hCFTR) mouse do not display a hyperinflammatory response to activation and, as such, fail to replicate a key aspect of human CF macrophage dysfunction. Heightened proinflammatory cytokine secretion in CF BMDMs in response to LPS has been reported in other CF mice including CFTR-nulls and models generated to mimic known clinical mutations (e.g. ΔF508, G551D) [18,20]. Following on from this, we find that macrophages derived from the *Cftr*^tm1Unc^ Tg(FABP-hCFTR) mouse display aberrant expression of the human *CFTR* transgene, which may underpin the absence of this CF-associated inflammatory phenotype. This is consistent with a previous report of human *CFTR* expression in alveolar macrophages derived from the same mouse, even in those that had been newly purchased prior to generations of inbreeding and the potential for genetic drift that accompanies that [21]. We also obtained bones from mice of the same model from a different institution and generated BMDMs as before, to find CFTR protein expression in macrophages derived from the CF mouse as well as WT littermates, therefore allowing us to conclude that this ectopic expression is likely present in all animals generated using this model, and was not simply a spontaneous, isolated occurrence. Furthermore, the observation that expression of human *CFTR* transgene in these BMDMs actually reduces the TNF response to LPS suggests that the presence of human WT *CFTR* may actually be anti-inflammatory in these cells. Highlighting this phenotype in BMDMs is particularly relevant to CF research, given that lineage-tracing studies have shown significant replenishment, or even complete replacement, of tissue-resident alveolar macrophages with blood-derived macrophages in the lung following infection or chronic inflammation [22], [23], [24], [25]. Consequently, it stands to reason that, in the context of severe CF lung disease, blood-derived macrophages likely become the predominant macrophage population responsible for mediating proper pulmonary resolution and repair. This has important implications for use of the *Cftr*^tm1Unc^ Tg(FABP-hCFTR) model. A better understanding of aberrant transgene expression in this mouse may also go some way to explaining certain anomalies in our understanding of macrophage function in CF more generally. Notably, the use of the gut-corrected Tg(FABP-hCFTR) CF mouse to propose *Cftr*-dependent regulation of phagosomal acidification as a key mechanism behind defective intracellular bacterial killing in CF macrophages was a major finding in the field, but spawned much controversy and has ultimately not proven reproducible despite the use of more accurate ratiometric methods with greater pH sensitivity than the fluorescein-based techniques employed in the original study [26], [27], [28]. One explanation for this ectopic transgene expression may be that murine macrophages express *Fabp2*. Although further investigation of this would be necessary for mechanistic confirmation, it is worth noting that expression of other FABPs has been shown previously in macrophages [1], [2], [3], [4], [5], [6], [7], [8], [9], [10], [11], [12], [13], [14], [15], [16], [17], [18], [19], [20], [21], [22], [23], [24], [25], [26], [27], [28], [29]. These findings suggest that, whilst the *Cftr*^tm1Unc^ Tg(FABP-hCFTR) mouse serves as a useful model of human disease and allows for drastically improved ease of husbandry relative the complete knockouts; other strains of CF mouse, such as ones harbouring human disease-specific mutations, should be considered for the study of myeloid cell dysfunction in CF.

## CRediT authorship contribution statement

**Jonathan L Gillan:** Conceptualization, Data curation, Methodology, Formal analysis, Investigation, Validation, Visualization, Writing – original draft, Writing – review & editing. **Gareth R Hardisty:** Conceptualization, Data curation, Methodology, Investigation, Visualization, Writing – review & editing. **Donald J Davidson:** Conceptualization, Resources, Writing – review & editing. **Robert D Gray:** Conceptualization, Funding acquisition, Resources, Writing – review & editing.

## Declaration of Competing Interest

None.
